# Interface Trap-Induced Temperature Dependent Hysteresis and Mobility in *β*-Ga_2_O_3_ Field-Effect Transistors

**DOI:** 10.3390/nano11020494

**Published:** 2021-02-16

**Authors:** Youngseo Park, Jiyeon Ma, Geonwook Yoo, Junseok Heo

**Affiliations:** 1 Department of Electrical and Computer Engineering, Ajou University, Suwon 16499, Korea; pys8685@ajou.ac.kr; 2 School of Electronic Engineering, Soongsil University, Seoul 06938, Korea; jiyeonma0@gmail.com

**Keywords:** *β*-Ga_2_O_3_, interface trap, hysteresis, mobility degradation, acceptor-like trap

## Abstract

Interface traps between a gate insulator and beta-gallium oxide (*β*-Ga_2_O_3_) channel are extensively studied because of the interface trap charge-induced instability and hysteresis. In this work, their effects on mobility degradation at low temperature and hysteresis at high temperature are investigated by characterizing electrical properties of the device in a temperature range of 20–300 K. As acceptor-like traps at the interface are frozen below 230 K, the hysteresis becomes negligible but simultaneously the channel mobility significantly degrades because the inactive neutral traps allow additional collisions of electrons at the interface. This is confirmed by the fact that a gate bias adversely affects the channel mobility. An activation energy of such traps is estimated as 170 meV. The activated trap charges’ trapping and de-trapping processes in response to the gate pulse bias reveal that the time constants for the slow and fast processes decrease due to additionally activated traps as the temperature increases.

## 1. Introduction

Beta-gallium oxide (*β*-Ga_2_O_3_) is a promising material for power semiconductors due to its superior electrical characteristics, such as a direct wide bandgap (4.6–4.9 eV) [1,2,3,4], a high electric breakdown field (~8 MV/cm) [5,6,7], a high electron saturation velocity (~2×10^7^ cm/s) [8], high carrier mobility (~100 cm^2^/V·s) [9,10,11], and thermal/chemical stability [12,13,14]. Furthermore, *β*-Ga_2_O_3_ exhibits the highest Baliga figure of merit (BFoM; defined as *εμE*_G_^3^, where *ε* is the dielectric constant, *μ* is the mobility, and *E*_G_ is the bandgap of the semiconductor) [15,16,17] among wide bandgap semiconductors: the BFoM represents a material parameter related to device power dissipation and the value of *β*-Ga_2_O_3_ is approximately ten times and four times higher than those of silicon carbide and gallium nitride, respectively [18,19].

Owing to these attractive characteristics, *β*-Ga_2_O_3_ has attracted much interest in a variety of potential applications such as high power transistors [5,7,10], chemical sensors [20], solar-blind ultraviolet (UV) detectors [13,21,22], UV astronomy, and space communication which require practical operation in harsh environments [12]. Thus, in recent times, doping methods [11,23], metal contacts [11,24], and large-area film deposition [9,25,26] of *β*-Ga_2_O_3_ have been actively investigated. In particular, the study of the interface characteristics between the gate insulator and the channel is of great significance because the charge trapping at the interface is a more fatal component in *β*-Ga_2_O_3_ field-effect transistors (FETs) than bulk traps, and consequently inhibits the high performance and reliability of the devices [27]. Therefore, understanding and controlling the defects at the interface is a critical step in the application of *β*-Ga_2_O_3_-based devices. Recent studies have reported on the observation of interface traps in *β*-Ga_2_O_3_ metal-oxide-semiconductor (MOS) capacitors and FETs using various gate insulators [8,27,28]. However, previous studies have not revealed the role of the interface traps in practical devices such as FETs or explicitly described their consequences on device operation.

Herein, we show that the interface trap induces hysteretic behavior in *β*-Ga_2_O_3_ FETs at high temperatures and also mobility degradations at low temperatures. To analyze the mobility degradation and hysteresis, a bottom-gate *β*-Ga_2_O_3_ FET was fabricated on SiO_2_ in this study. The trapping/de-trapping processes at the interface between *β*-Ga_2_O_3_ and SiO_2_ and their effects on the mobility in the *β*-Ga_2_O_3_ FET were studied by analyzing temperature-dependent electrical characteristics in the temperature range of 20–300 K. Additionally, trap-related parameters including the activation trap energy of the interface traps and the trapped charge densities were extracted by means of temperature-dependent hysteresis and transient analysis. This work will expand our understanding of the temperature-dependent characteristics and physical origin of the trap charges in *β*-Ga_2_O_3_ FETs.

## 2. Experiments

### 2.1. Device Fabrication

The (−201) surface *β*-Ga_2_O_3_ bulk substrate with unintentional n-type doping was purchased from Tamura Corporation, Japan. Multi-layer *β*-Ga_2_O_3_ flakes were mechanically transferred using a conventional scotch tape method from the *β*-Ga_2_O_3_ bulk substrate onto a heavily doped p-type Si substrate with thermally grown 300 nm SiO_2_. The source and drain electrodes were defined on top of the Ga_2_O_3_ flakes by photolithography, 20/100 nm Ti/Au electron-beam evaporation, and a conventional lift-off process. The fabricated device was annealed at 450 °C in nitrogen ambient for 1 min using a rapid thermal process to improve contact resistance.

### 2.2. Temperature-Dependent Electrical Measurements

The fabricated device was mounted in a liquid helium closed-cycle cryostat (Janis Research, CCS-150, Woburn, MA, USA). Temperature-dependent electrical characteristic measurements were carried out in a high vacuum (<10^−3^ Pa) with a semiconductor parameter analyzer (Keithley, 4200A-SCS, Solon, OH, USA) to identify the intrinsic effects without ambient environmental effects like water and oxygen molecules. To obtain the transfer curves, the gate bias (*V*_GS_) was swept from −40 to 10 V (forward sweep) and then back to −40 V (backward sweep) while maintaining drain bias (*V*_DS_) values of 0.5 and 1 V. The output characteristics were measured by sweeping *V*_DS_ from 0 to 10 V while varying the *V*_GS_ (−15, −10, −5, 0, 5, and 10 V). The transient response was measured by the alternate *V*_GS_ at a fixed *V*_DS_ of 1 V. To reach equilibrium, we first applied *V*_GS_ of 10 V for 600 s until *I*_DS_ was saturated. Gate pulses were then changed from 10 V to 0 V and maintained for 150 s, and vice versa.

### 2.3. Contact Resistance, Mobility, and Threshold Voltage Extraction

The contact resistance (*R*_C_) was calculated as follows: *R*_C_ = *L*·*θ/*(*W*·*C*_OX_·*μ*_0_), where *θ* is the effective mobility attenuation factor, *W* is the channel width, *L* is the channel length, *C*_OX_ is the capacitance of the SiO_2_, and *μ*_0_ is the low-field mobility [29]. The low-field mobility was extracted from the Y-function, given by *Y* = (*μ*_0_·*C*_OX_·*V*_DS_·*W*/*L*)^0.5^(*V*_GS_ – *V*_th,Y_), where *V*_th,Y_ was the threshold voltage extracted from Y-function. The effective mobility attenuation factor was extracted as follows:

(1) $$I_{\mathrm{DS}}=\left(\frac{\mu 0}{1\;+\;\theta \left(V_{GS}\;–\;V_{th,Y}\right)}\;\right)C\mathrm{ox}\frac{W}{L}\left(V_{GS}\;–\;V_{th,Y}\right)V_{DS}$$

We extracted the contact resistance and the mobility from the same transfer curve measured at *V*_DS_ = 1 V. The mobility was extracted from the transfer characteristics. To observe the mobility except for the effect of contact resistance, we first extracted the actually applied drain voltage in the channel (*V*_DS,CH_) as follows: *V*_DS,CH_ = *V*_DS_ – *R*_C_ × *I*_DS_. Subsequently, the field-effect mobility was calculated as *μ*_FE_ = *L*·*g*_m_/(*W*·*C*_OX_·*V*_DS,CH_), where *g*_m_ is the transconductance. We estimated the threshold voltage as the *x*-intercept of the tangential line at the maximum slope on the transfer curve on a linear scale (Figure S1 in Supplementary Materials).

### 2.4. The Interface Trap Density, the Amount of Charge, Time-Dependent Trapped Charge Density Changes, and Trap Parameter Extraction

The interface trap density is estimated from *SS* = ln(10)·*kT*/*q*·(1+(*C*_S_+*C*_it_)/*C*_OX_), where *SS* is the subthreshold swing, *C*_S_ is the capacitance of *β*-Ga_2_O_3_ conducting channel and *C*_it_ = *q*^2^ × *D*_it_ is the capacitance induced by the interface trap density [4,30]. The amount of charge trapped and de-trapped by the interface trap is extracted as Δ*Q*_hy_ = *C*_OX_ × Δ*V*. To determine the activation energy of the interface trap, Δ*Q*_hy_ was fitted by Δ*Q*_hy_ = *Q*_m_ × exp(−*E*_A_/*k*_B_*T*) + *Q*_fix_, where *Q*_m_ is the maximum charge density, *k*_B_ is the Boltzmann constant, and *Q*_fix_ is the temperature-independent fixed charge density [30].

The time-dependent density of the trapped charges changes is expressed as *Q*_it_(t) – *Q*_it_(0) = – (*I*_DS_(t) – *I*_DS_(0))*ε*·*ε*_0_/*q*·*t*_OX_·*g*_m_, where *Q*_it_(t) is the density of trapped charges (*Q*_it_) as a function of time, *Q*_it_(0) is *Q*_it_ at the time changed the gate pulses, *I*_DS_(t) is *I*_DS_ as a function of time, *I*_DS_(0) is *I*_DS_ at the edge of gate pulses, *ε* is the relative dielectric constant of the oxide, *ε*_0_ is the permittivity of vacuum, and *t*_OX_ is the thickness of the oxide. The transient *Q*_it_(t) – *Q*_it_(0) is fitted with a bi-exponential equation: *Q*_it_(t) – *Q*_it_(0) = *Q*_1_·exp(–*t*/*τ*_it1_) + *Q*_2_·exp(–*t*/*τ*_it2_), where *Q*_1_ and *Q*_2_ are the density of trapped charges and *τ*_it1_ and *τ*_it2_ are the time constants [26].

## 3. Results and Discussion

**Figure 1:**
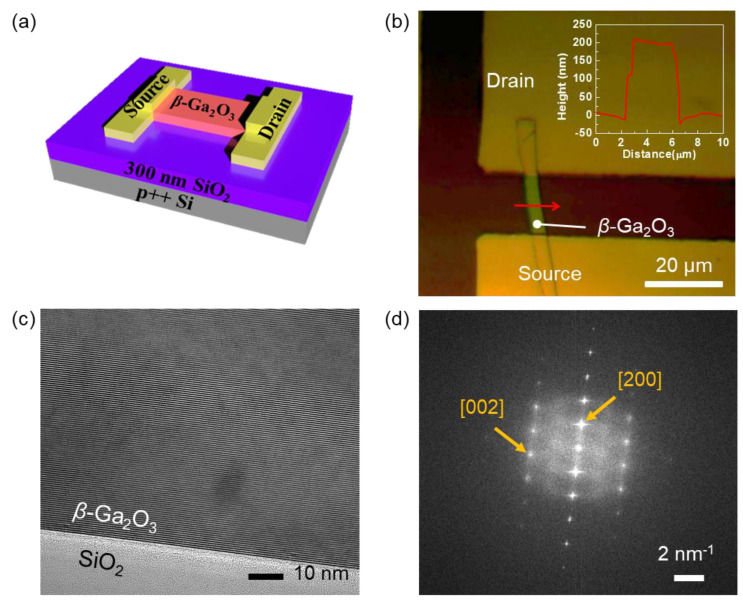
(**a**) Schematic of the *β*-Ga_2_O_3_ field-effect transistor, (**b**) Optical microscopy image of the fabricated field-effect transistor. The *β*-Ga_2_O_3_ channel thickness along the red line was characterized by atomic force microscopy, and the profile is shown in the inset. (**c**) Cross-sectional HR-TEM image of the interface between *β*-Ga_2_O_3_ channel and SiO_2_ insulator. (**d**) Selective area electron diffraction pattern of the *β*-Ga_2_O_3_.

The schematic in Figure 1a shows the fabricated *β*-Ga_2_O_3_ FET on a heavily doped p-type Si substrate as the bottom gate with SiO_2_ gate insulator. SiO_2_ is potentially of a higher dielectric reliability in FETs due to the larger conduction band offset between SiO_2_ and Ga_2_O_3_ compared to Al_2_O_3_ and Ga_2_O_3_ [28]. Figure 1b shows an optical microscope image of the fabricated *β*-Ga_2_O_3_ device, and we measured *W* as 3.3 μm and *L* as 15 μm. A thickness of the *β*-Ga_2_O_3_ channel layer was measured as approximately 200 nm using atomic force microscopy (the inset in Figure 1b). A cross-sectional high resolution transmission electron microscopy (HR-TEM) image in Figure 1c represents the smooth interface between *β*-Ga_2_O_3_ and SiO_2_. Mechanical exfoliation of *β*-Ga_2_O_3_ and subsequent transfer on SiO_2_ did not result in any damage or defect, and a high quality of crystalline was preserved in the fabricated device. The selective-area diffraction pattern was also characterized as shown in Figure 1d. [200] and [002] directions in monoclinic crystal structure were indicated, and the channel surface was confirmed as the *β*-Ga_2_O_3_(100) plane.

**Figure 2:**
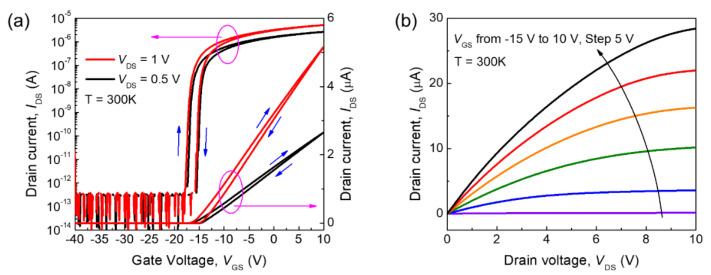
Characteristics of the fabricated *β*-Ga_2_O_3_ field-effect transistor: (**a**) transfer characteristics for *V*_DS_ = 0.5 (black line) and 1 V (red line) at room temperature (*V*_GS_ was swept from –40 to 10 V (forward sweep) and then back to –40 V (backward sweep)), and (**b**) output characteristics for *V*_GS_ = –15, –10, –5, 0, and 5 V at room temperature of the fabricated *β*-Ga_2_O_3_ field-effect transistor.

The fabricated *β*-Ga_2_O_3_ FET was characterized by measuring its current-voltage characteristics. Figure 2a,b presents the measured transfer and output characteristics at room temperature, respectively. The fabricated device operates in depletion mode. We extracted the mobility, subthreshold swing, and threshold voltage of the device at room temperature from the transfer characteristics. At *V*_DS_ = 1 V, maximum *μ*_FE_ of the *β*-Ga_2_O_3_ FET were 83.5 and 88.3 cm^2^/V·s, *SS* were 180 and 130 mV/dec, and threshold voltages (*V*_th_) were −15.5 and −14 V for forward and backward sweeps, respectively, and an ON/OFF ratio (*I*_ON_/*I*_OFF_) of approximately 10^7^ was observed. Hysteresis (~1.5 V), a threshold voltage difference in the transfer curves depending on the sweep directions, was observed. The *D*_it_ was estimated to be approximately 1.44 × 10^11^ cm^–2^eV^–1^ from *SS* at 300 K. The *D*_it_ extracted in this work is similar to the previously other reports [27,28]. We also confirmed Ohmic contact behaviors from good linearity of the output curves near 0 V (Figure S2 in Supplementary Materials).

**Figure 3:**
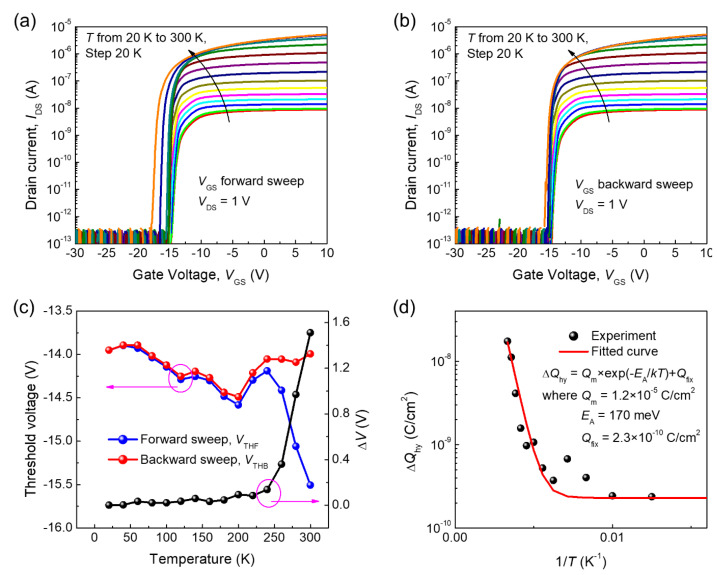
Transfer characteristics of the fabricated *β*-Ga_2_O_3_ field-effect transistor: (**a**) forward sweep and (**b**) backward sweep at various temperatures from 20 to 300 K, (**c**) the temperature-dependent threshold voltage in the forward (blue) and backward (red) sweeps and the temperature-dependent degree of hysteresis (black; the difference between the threshold voltages in the forward and backward sweeps), and (**d**) Arrhenius plots of the Δ*Q*_hy_ trapped and de-trapped by the interface traps (the red solid line is the best fit of Δ*Q*_hy_).

The temperature-dependent hysteretic behaviors of two-dimensional materials were previously reported by our group [30], and in general, it is prevalent that the interface traps and associated charges are responsible for it. Therefore, to investigate the origin of the hysteresis in the *β*-Ga_2_O_3_ FET in detail, its temperature dependence was characterized. Figure 3a,b presents the respective transfer curves for forward and backward sweeps at various temperatures from 20–300 K, and two properties were observed from the temperature-dependent transfer curves. First, the drain on-current (above the threshold) decreased by approximately three orders of magnitude as the temperature varied from 300 to 20 K. Decreasing the temperature would have contributed to the mobility decrease, and a detailed discussion on this follows later on. Second, as the temperature increased, the threshold voltage in the forward sweep shifted more toward a negative value than the one in the backward sweep. In other words, the hysteresis increased as the temperature increased.

**Figure 4:**
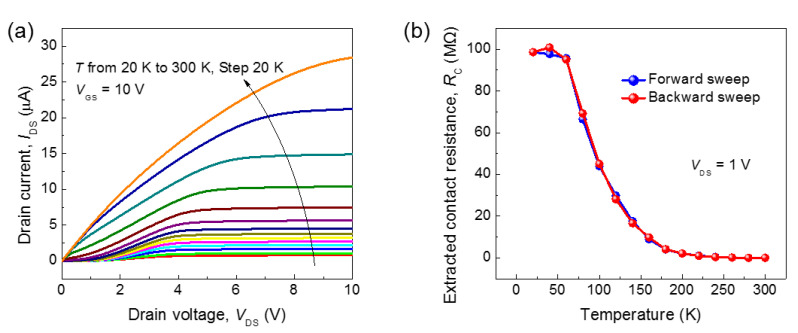
(**a**) Output characteristics at a gate bias (*V*_GS_) of 10 V in the temperature range of 20–300 K and (**b**) extracted contact resistance at *V*_DS_ = 1 V of the fabricated *β*-Ga_2_O_3_ field-effect transistor in the forward (blue) and backward (red) sweeps.

To analyze the hysteresis, we extracted the threshold voltage from the temperature-dependent transfer curves. Figure 3c shows the variations of the *V*_th_ (left) in the forward (*V*_THF_, blue) and backward (*V*_THB_, red) sweeps. For consistency, we applied the same method to extract the threshold voltages for different temperatures. Thus, despite the monotonic left-shift of the transfer curves as the temperature increased, the additional change of slope on a linear scale made the variations of the extracted threshold voltage look more or less random and uncorrelated with temperature. Since the degree of hysteresis (Δ*V*) is defined as the difference between *V*_THF_ and *V*_THB_, any artifact made while obtaining an individual threshold voltage would be canceled out. As shown in Figure 3c, the variations in Δ*V* below 230 K were almost negligible but started to increase at around 230 K and rose sharply above 230 K due to the thermal activation of the interface traps and the associated charges responsible for the hysteresis. For a more quantitative analysis, the Δ*Q*_hy_ trapped and de-trapped by the interface trap was extracted and then fitted by an Arrhenius plot, as shown in Figure 3d. The best fit of Δ*Q*_hy_ was obtained with the fitting parameter values of *Q*_m_ = 1.2×10^−5^ C/cm^2^, *E*_A_ = 170 meV, and *Q*_fix_ = 2.3×10^−10^ C/cm^2^ (see the Methods section for more detail). The interface trap is partially active at room temperature even if the activation energy of 170 meV is six to seven times greater than the thermal energy at room temperature. *Q*_fix_, the charge density irrespective of the temperature, is five orders of magnitude smaller than *Q*_m_. In other words, most of the interface trap charges responsible for the hysteresis are governed by the temperature. In addition to the temperature-dependent hysteresis, the drain current (*I*_DS_) decreased in the transfer curve because the mobility is reduced with decreasing temperature, and this temperature-dependent current drop was also observed in the output characteristics. As shown in Figure 4a, the saturation *I*_DS_ decreased and the slope in the linear region decreased with decreasing temperature, due to an increase in contact resistance (*R*_C_) and a decrease in mobility. As can be seen, the *I*_DS_ no longer showed linear dependence on the bias near the low *V*_DS_ as the temperature decreased, and the *R*_C_ values were no longer negligible at low temperature. We extracted the *R*_C_ from the modified Ghibaudo Y-function method [29] and the mobility by considering the finite *R*_C_ (see the Methods section for more details). The temperature-dependent *R*_C_ was calculated for forward and backward sweeps of the transfer curve, as shown in Figure 4b. *R*_C_ was 6.3 kΩ at 300 K and then rapidly increased up to 100.8 MΩ as the temperature decreased, possibly because of the reduced thermal energies of carriers for thermionic emission over the Schottky barrier.

**Figure 5:**
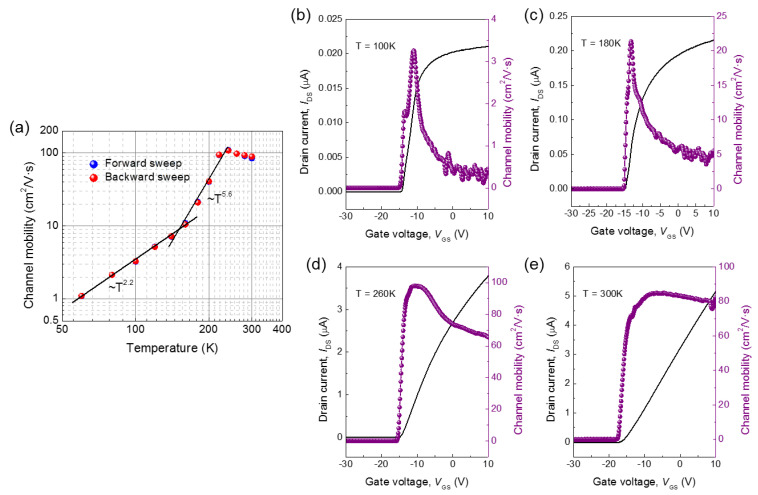
(**a**) Maximum channel mobilities at various temperatures (*T* = 60–300 K) on a log-log scale (the black solid lines are the fitted channel mobilities proportional to approximately *T*^2.2^ (*T* = ~150 K) and *T*^5.6^ (*T* = 150–230 K)) and (**b**–**e**) transfer curves on a linear scale for *T* = 100, 180, 260, and 300 K with the extracted channel mobilities of the fabricated *β*-Ga_2_O_3_ field-effect transistor.

The maximum channel mobilities (*μ*_CH_) at various temperatures considering the effect of the contact resistance are plotted on a log-log scale in Figure 5a. It is worth noting that the channel mobility steeply decreased as the temperature decreased below 230 K. At low temperature (generally below 100 K), the mobility reduced due to the impurity scattering and became proportional to *T*^γ^ (*γ* = 1.5) as the reported Hall mobility [23,25,31,32] but here it decreased more quickly with *γ* at approximately 2.2 (*T* = ~150 K) and 5.6 (*T* = 150–230 K). Thus we cannot explain this mobility degradation by the impurity scattering, and the surface related scattering would be responsible for it. In Figure 5b–e, the transfer curves at *T* = 100, 180, 260, and 300 K are plotted on a linear scale, respectively, and the extracted channel mobilities in each are also shown alongside. Implausibly, *I*_DS_ at 100 K rapidly saturated at just above the threshold whereas that at 300 K increased linearly. That is to say, the channel mobility became less affected by the gate bias as the temperature increased. Interestingly, the channel mobility started to decrease below 230 K (the temperature that coincides with the hysteresis becoming invisible). Therefore, these trends contributed to the interplay between interface scattering and the effect of acceptor-like traps at the interface between *β*-Ga_2_O_3_ and SiO_2_ [27]. At temperatures below 230 K, the acceptor-like traps were frozen and electrically neutral, allowing more electrons to drift along the vicinity of the interface. The interface scattering became predominant over any other scatterings including ionized impurity scattering, and mobility steeply decreased. This can also be envisioned by the strong gate bias dependence of the channel mobility at low temperature, as discussed earlier (Figure 5b), and the previous reports of strong mobility degradation by the surface effects in *β*-Ga_2_O_3_ thin-films at low temperatures [33].

**Figure 6:**
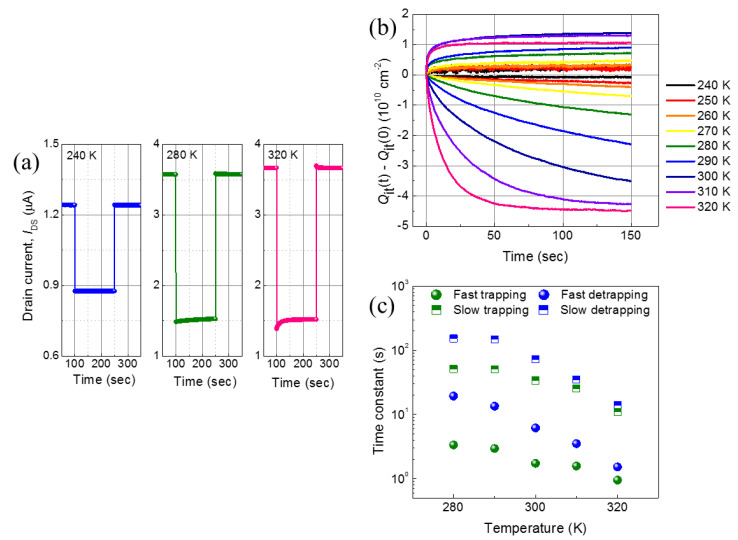
(**a**) Transient responses of *I*_DS_ to the *V*_GS_ changes at *T* = 240, 280, and 320 K, (**b**) changes of trapped charge density as a function of time from 240 to 320 K, and (**c**) the extracted temperature-dependent time constants for the slow and fast processes of the fabricated *β*-Ga_2_O_3_ field-effect transistor.

We also carried out a time-domain analysis on the capture and release of charges by the traps at the interface by measuring the transient response to observe the behaviors of the traps [34]. As shown in Figure 6a, as soon as *V*_GS_ dropped, *I*_DS_ sharply dropped and then slowly increased because the captured carriers were released from the interface traps. At the rising edge of *V*_GS_, *I*_DS_ popped up and then slowly decreased while maintaining *V*_GS_ because of the electrons captured by the acceptor-like traps at the interface. As the temperature increased, these phenomena noticeably appeared because more of the acceptor-like traps were activated. We calculated the transient changes of the trapped charge density for the temperature from 240 to 320 K, as shown in Figure 6b; as the temperature increased, the changes of trapped and de-trapped charge density also increased. The transient changes were not observed below 240 K because of negligible variation by the trapped charge. Figure 6c shows temperature-dependent time constants extracted by fitting Figure 6b with a bi-exponential equation [35]. All of the time constants for the slow and fast processes decreased as the temperature increased because the activated traps were more abundant and the electrons were more thermally energized. We also observed that the charge trapping processes were faster than the charge de-trapping processes. The detailed time constants are available in the Supplementary Materials (Table S1).

## 4. Conclusions

In summary, we investigated the effect of interface traps on the degradation of channel mobility and hysteresis in a bottom-gated *β*-Ga_2_O_3_ FET at low temperature. Temperature-dependent electrical characterizations were performed on the device in the temperature range of 20–300 K, and variations in threshold voltage and field-effect mobility and the degree of hysteresis were analyzed. The activation energy of the interface trap between *β*-Ga_2_O_3_ and SiO_2_ was estimated as 170 meV, and there was no observable hysteresis below 230 K. As the acceptor-like traps at the interface are frozen and inactive at low temperature, the hysteresis disappears and it was simultaneously found that the channel mobility sharply decreases. This was understood as the frozen charged traps allow the channel electrons to collide at the interface, which was also confirmed by the vulnerability of mobility to gate bias at low temperature. Furthermore, the charge trapping and de-trapping processes at the interface were studied in the time-domain by switching the gate bias. At higher temperatures, the extracted time constants for the slow and fast processes became shorter due to more activated traps. We believe that understanding the role of the interface traps between the gate insulator and *β*-Ga_2_O_3_ could help to optimize the fabrication and operation of *β*-Ga_2_O_3_-based devices in a variety of circumstances, particularly in harsh environments in space and military applications.

## Data Availability

The data presented in this study are available on request from thecorresponding author.

## Supplementary Materials

The following are available online at https://www.mdpi.com/2079-4991/11/2/494/s1, Figure S1. Transfer curve for *V*_DS_ = 1 V in a linear scale at 180 K, Figure S2. Output curves of *I*_DS_-low *V*_DS_ at room temperature, Table S1. The density of the trapped and de-trapped charges and the time constants.
