# Supplementary material for: Interface Trap-Induced Temperature Dependent Hysteresis and Mobility in β-Ga2O3 Field-Effect Transistors
Source: Nanomaterials (Basel). 2021 Feb 16;11(2):494. doi: 10.3390/nano11020494 (PMC7920063; doi:10.3390/nano11020494)
Supplement: Supplementary file 1 [file nanomaterials-11-00494-s001.pdf]

Supporting Information

Interface Trap-Induced Temperature Dependent Hysteresis and Mobility in β-Ga_2O_3 Field-Effect Transistors

Youngseo Park [1], Jiyeon Ma [2], Geonwook Yoo [2,*] and Junseok Heo [1,*]

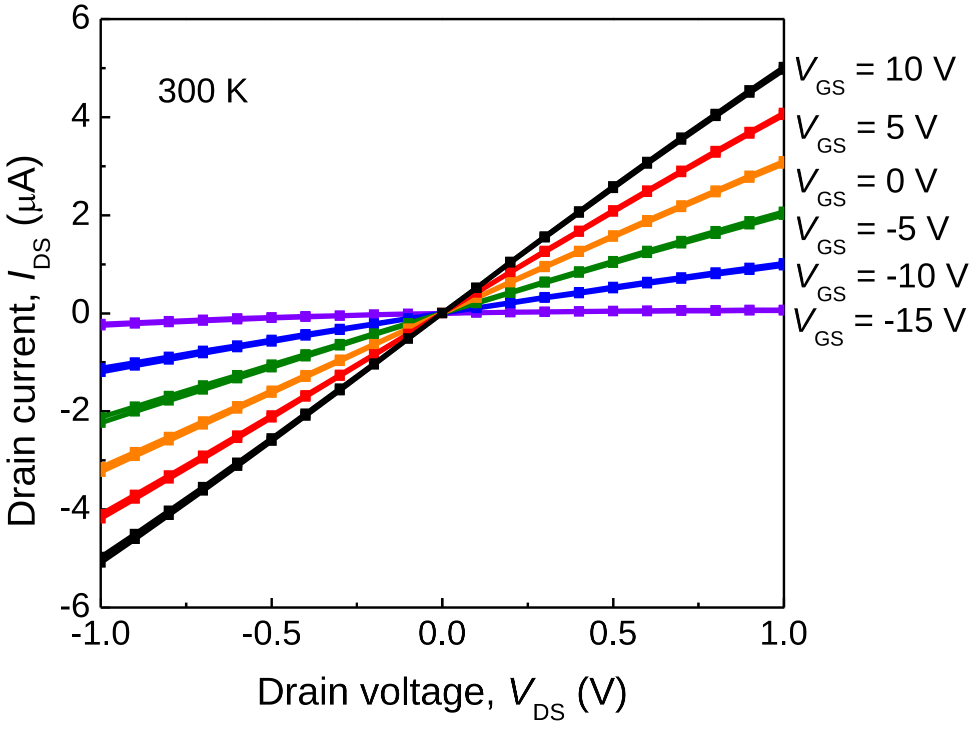

Figure S1. Output curves of I_{DS} – low V_{DS} at room temperature for V_{GS} = -15, -10, -5, 0, 5, and 10 V. The good linearity of output curves indicate the Ohmic contact of the β-Ga_2O_3 FET.

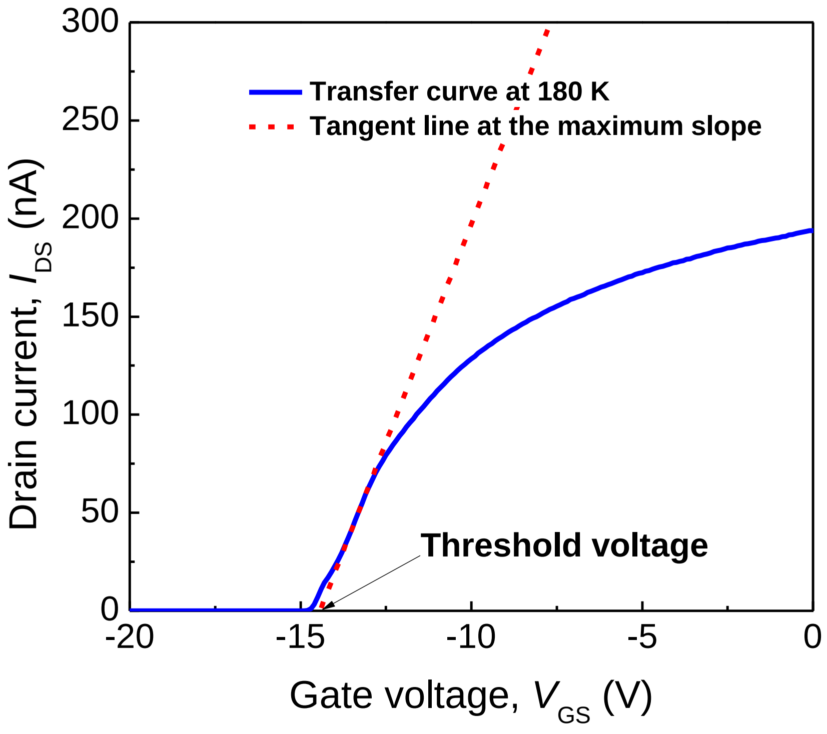

Figure S2. Transfer curve for V_{DS} = 1 V in a linear scale at 180 K. The red dash line is tangential line at the maximum slope on transfer curve. The threshold voltage is defined as x-intercept of the red dash line.

Table S1. The density of the trapped and de-trapped charges and the time constants.

$$I = I_0 \pm \frac{\mu W V_{DS}(Q_1 e^{-t/\tau_{it1}} + Q_2 e^{-t/\tau_{it2}})}{L}$$

	V_G = 0 V					V_G = 10 V				
**T [K]**	Q_1+Q_2 [10^9 cm^{-2}]	Q_1 [10^9 cm^{-2}]	τ_{it1} [s]	Q_2 [10^9 cm^{-2}]	τ_{it2} [s]	Q_1+Q_2 [10^9 cm^{-2}]	Q_1 [10^9 cm^{-2}]	τ_{it1} [s]	Q_2 [10^9 cm^{-2}]	τ_{it2} [s]
**280**	19.23	2.33	19.41	16.9	153.13	-5.53	-2.52	3.35	-3.01	51.04
**290**	33.18	4.14	13.44	29.04	148.44	-7.73	-3.55	2.95	-4.18	50.72
**300**	38.61	4.77	6.16	33.84	72.24	-11.06	-6.09	1.73	-4.97	34
**310**	42.56	6.63	3.51	35.93	34.86	-10.71	-6.55	1.57	-4.16	25.35
**320**	42.7	5.84	1.52	36.86	13.89	-9.61	-5.69	0.95	-3.92	10.96
